# Identification of Epigenetic Regulators of a Transcriptionally Silenced Transgene in Maize

**DOI:** 10.1534/g3.111.000232

**Published:** 2011-06-01

**Authors:** Thelma F. Madzima, E. Shannon Mills, Jack M. Gardiner, Karen M. McGinnis

**Affiliations:** Department of Biological Science, Florida State University, 319 Stadium Drive, Tallahassee, Florida 32306-4295

**Keywords:** epigenetics, transgene, gene silencing, genetic screen

## Abstract

Transcriptional gene silencing is a gene regulatory mechanism essential to all organisms. Many transcriptional regulatory mechanisms are associated with epigenetic modifications such as changes in chromatin structure, acetylation and methylation of core histone proteins, and DNA methylation within regulatory regions of endogenous genes and transgenes. Although several maize mutants have been identified from prior forward genetic screens for epigenetic transcriptional silencing, these screens have been far from saturated. Herein, the transcriptionally silent *b1* genomic transgene (*BTG*-silent), a stable, epigenetically silenced transgene in *Zea mays* (maize), is demonstrated to be an effective phenotype for a forward genetic screen. When the transgene is reactivated, a dark purple plant phenotype is evident because the B1 transcription factor activates anthocyanin biosynthesis, making loss of silencing mutants easy to identify. Using *BTG*-silent, ten new putative mutants were identified and named *transgene reactivated1* through *11* (*tgr1-6* and *tgr8-11*). Three of these mutants have been examined in more detail, and molecular and genetic assays demonstrated that these mutants have both distinct and overlapping phenotypes with previously identified maize mutants that relieve epigenetic transcriptional silencing. Linkage analysis suggests that *tgr2* and *tgr3* do not correspond to a mutation at previously identified maize loci resulting from other forward genetic screens, while *tgr1* shows linkage to a characterized gene. These results suggest that the mutants are a valuable resource for future studies because some of the mutants are likely to reveal genes that encode products required for epigenetic gene regulation in maize but are not currently represented by sequenced mutations.

Transcriptional regulation of gene expression is essential for the normal growth and development of organisms. Transcriptional regulation is accomplished via the association of transcription factors with genetic regulatory elements nearby or adjacent to the regulated gene and transcription factor accessibility to those sequences, which is dependent on the chromatin structure. In eukaryotic species, the establishment and maintenance of a particular conformation of chromatin involves an interdependent association of differentially methylated DNA, modified histones, and variations in nucleosome distribution and compaction (reviewed by Goldberg *et al.* 2007). A particular chromatin structure and its associated gene expression state is sometimes heritable across cell divisions, contributing to cellular differentiation and development (Jarillo *et al.* 2009).

The methylation of cytosines in a symmetric CG context is highly conserved in plants and mammals and is generally considered to be associated with the regulation of gene expression, although the exact nature of the relationship between gene expression and DNA methylation is not completely understood (reviewed by Lee *et al.* 2010). In plants, cytosine methylation within gene promoters, repetitive sequences and transposons can be associated with transcriptional gene silencing (TGS) via the RNA-dependent DNA methylation (RdDM) pathway and can involve asymmetric cytosine residues (CHH, where H is any residue other than G) in addition to symmetric cytosine residues (CG, CHG). This pathway is reliant upon the activity of a set of proteins that produce small interfering RNAs (siRNAs) homologous to target loci and mediate epigenetic features on the chromosome at target loci (reviewed by Matzke *et al.* 2009; Simon and Meyers 2010). Although RdDM has been characterized most extensively in Arabidopsis, genetic screens in maize have identified several components of the pathway and the maize orthologs have been implicated in the regulation of both endogenous and transgenic loci, including those that participate in paramutation (reviewed by Arteaga-Vazquez and Chandler 2010).

Paramutation occurs when two alleles interact in *trans* to heritably change the expression level of one allele (reviewed by Chandler 2010). Paramutation in maize has been described at several genes involved in the flavonoid biosynthetic pathway and include *r1*, *b1*, *pl1*, and *p1* (Brink 1956; Coe 1959; Hollick *et al.* 1995; Sidorenko and Peterson 2001) (reviewed by Chandler *et al.* 2000). Each of these genes encodes a transcription factor that regulates the expression of the enzymes required for pigment biosynthesis, allowing transcriptional activity of these genes to be readily detected by the visual observation of pigment in plant tissues. At the *b1* gene, paramutation requires the presence of tandem hepta-repeat sequences located 100 kb upstream of the *b1* transcription start site (reviewed by Chandler 2010). This well-characterized example of paramutation involves transcriptional silencing of one allele, associated with changes in DNA methylation and chromatin structure within the tandem repeats (Haring *et al.* 2010; Stam *et al.* 2002a; Stam *et al.* 2002b).

The *b1* and *pl1* systems have been used in genetic screens to identify mutants required for paramutation. Using either active *Mutator* (*Mu*) transposable elements or EMS mutagenesis, genetic screens based on these paramutation systems have identified several mutants. The *b1* paramutation alleles were used to identify the *mediator of paramutation* (*mop*) mutants (Dorweiler *et al.* 2000; Sidorenko *et al.* 2009). Map-based cloning revealed that the first locus characterized, *mop1*, encodes a putative RNA-dependent RNA-polymerase similar to Arabidopsis RDR2 (Alleman *et al.* 2006). More recently, a dominant mutation designated *Mop2-1* was cloned and shown to encode a protein similar to the second largest subunits of Pol IV and Pol V in Arabidopsis (Sidorenko *et al.* 2009). The *pl1* paramutation system was used to identify several mutants designated *required to maintain repression* (Hale *et al.* 2007; Hollick and Chandler 2001; Stonaker *et al.* 2009). *rmr1* encodes a putative SNF2-like ATPase chromatin remodeler (Hale *et al.* 2007) and *rmr6* encodes a protein similar to Arabidopsis NRPD1, which is the large subunit of the plant specific DNA-dependent RNA polymerase (Pol IV) (Erhard *et al.* 2009). *rmr7* encodes an allele of *mop2* (Stonaker *et al.* 2009). Identification and cloning of these genes provides strong evidence that RNA-directed transcriptional gene silencing is one mechanism underlying paramutation. While the endogenous *b1* and *pl1* systems have been useful in the discovery of genes required for both paramutation and TGS, many of the maize orthologs for Arabidopsis RdDM mutants have yet to be identified (reviewed by Arteaga-Vazquez and Chandler 2010). Approaching gene discovery through multiple screens will enhance the likelihood of characterizing as many components of epigenetic gene regulation as possible in this important model organism.

The *mop1-1*, *rmr1-1*, and *rmr2-1* mutants were identified in genetic screens for paramutation, and *Mop1*, *Rmr1*, and *Rmr2* are also required for epigenetic silencing of two transgenes (McGinnis *et al.* 2006), including the *b1* genomic transgene (BTG). This transgene includes the maize *b1* genomic sequence (transcribed region including introns and exons) driven by the heterologous, highly expressed 35S cauliflower mosaic virus promoter (Figure 1A); plants actively expressing this transgene are purple. A stably silent line was identified (*BTG*-silent) in which the plant tissues were green due to transcriptional silencing of the transgene, even when the transgene remained active in the kernel (McGinnis *et al.* 2006). Because none of the sequences required for *b1* paramutation are included in the transgene (reviewed by Chandler 2010), and all of the described experiments used maize stocks that did not carry *b1* alleles that participate in paramutation, the transcriptional silencing of this transgene is not directly related to *b1* paramutation. *BTG*-silent provides a powerful marker for investigating epigenetic gene silencing and a system in which heritable changes in gene expression can be correlated with specific epigenetic marks and mechanisms (McGinnis *et al.* 2006).

**Figure 1:**
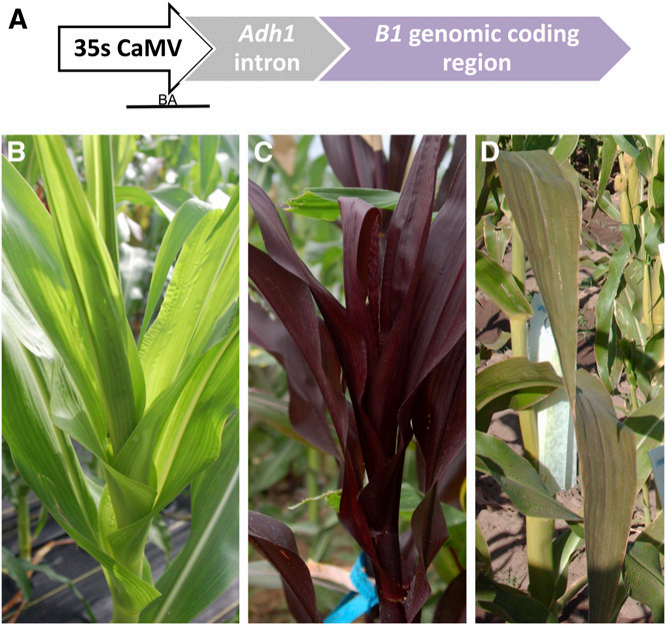
Phenotypes of plants transgenic for the *b1* genomic transgene. (A) The b1 genomic transgene includes the 35S Cauliflower Mosaic Virus promoter (35SCaMV), the first intron of the maize *Adh1* gene (Adh1 intron) to enhance expression, and the coding region of B1 (B1 genomic coding region). The region of the transgene analyzed by bisulfite sequencing is indicated (BA). (B) In nonmutant plants, the transgene is transcriptionally silent (*BTG*-silent), and there is no observable anthocyanin pigmentation in most plant tissues. (C) In the majority of the *tgr* mutants (with the exception of *tgr3*), the transgene is strongly reactivated, and transcription of BTG results in dark pigment in most above ground tissues (*BTG*-active). This phenotype was exhibited by *tgr1*, *tgr2*, *tgr4*, *tgr5*, *tgr6*, *tgr8*, *tgr9*, *tgr10*, and *tgr 11*; the plant shown is *tgr1*. (D). In *tgr3* individuals, BTG-active has a much lower level of pigment in plant tissues relative to the other mutants.

To identify other genes involved in epigenetic gene regulation, a forward genetic screen based on reactivation of *BTG*-silent was conducted using EMS mutagenized maize. Reactivation of the transgene is easily scored by visually monitoring accumulation of anthocyanin pigmentation. Herein, we report that the *BTG*-silent transgene and EMS mutagenesis were effectively used to identify multiple genes required for transcriptional silencing of transgenes in maize; these genes are referred to as *transgene-reactivated* (*tgr*). Initial genetic and molecular characterizations of these mutants are reported.

## Materials and Methods

### Genetic stocks and plant material

The *b1* genomic transgenic line has been described previously (McGinnis *et al.* 2006). Briefly, the line is transgenic for the 35SBTG construct, which is composed of the 35S Cauliflower Mosaic Virus promoter (35S CaMV), the first intron of maize *alcohol dehydrogenase1* (included as an enhancer of expression), and the genomic sequence spanning the complete coding region and both the 5′ and 3′UTRs of the *B-I* allele of the maize *b1* gene (McGinnis *et al.* 2006) (Figure 1A). *B-I* encodes a transcription factor which activates anthocyanin biosynthetic genes, leading to red or purple plant pigmentation in tissues where it is expressed (Selinger *et al.* 1998). Following transformation of the maize inbred line CG00526 with the 35SBTG construct, the transgenic line has been crossed for many generations with stocks that were recessive for *b1* and the functionally redundant *r1* locus, wild-type for the other transcriptional regulators and wild-type for all the biosynthetic enzymes required for pigment production. In this line, BTG is stably, heritably, and transcriptionally silenced (McGinnis *et al.* 2006). The chromosomal location of BTG is unknown, but the same transgenic event was used for all experiments described herein. Details on the genotype of this stock are available upon request.

### Mutagenesis and forward genetic screen

Mutagenesis was conducted by treatment of nontransgenic pollen with ethyl methanesulfonate (EMS) (Neuffer *et al.* 1997). EMS was diluted in paraffin oil and applied to pollen collected from nontransgenic *b r-g* stocks. After an incubation period, treated pollen was applied to ears of plants hemizygous or homozygous for *BTG*-silent. Ears were covered and allowed to mature for the remainder of the field growing season. Resulting seeds (the M_1_ generation) were planted and self-pollinated to make any introduced mutations homozygous. The M_1_ generation was monitored for increased pigment in plant tissues, which could be indicative of dominant or semidominant alleles that reactivated the transgene—none were observed. After self-pollination, 60 transgenic M_2_ seeds were planted (McGinnis *et al.* 2006). Approximately 500 M_2_ families were screened, resulting in 10 putative recessive mutants identified. One M_2_ family was originally designated as *tgr7*, but it only segregated a very small number of dark plants, and no dark plants segregated upon replanting. This line was dropped from analysis; consequently, there is no putative mutant corresponding to *tgr7*.

### Nomenclature

Putative mutants from this screen were designated *tgr* for *transgene reactivated*. For each individual, two independent loci are relevant for the experiments, one is the putative mutated locus (*tgr1* through *tgr11*), and the other is the transgene (*BTG*-silent/active). Transgenic lines are designated by their *tgr* family number and the activation status of *BTG*. For example, a given family, which results from planting seed from a single ear, will be segregating wild-type and mutant *tgr* alleles and segregating the transgene, which will be silent or active. Genotyping is not possible as the mutated loci are as yet uncloned, but we refer to the various combinations of loci using the hypothesis that plants bearing silent transgenes are heterozygous for the recessive mutant or homozygous wild-type, while plants with active transgenes are homozygous for the mutation.

### Bisulfite conversion and DNA methylation analysis

Genomic DNA was isolated from adult leaf tissue using the DNeasy Plant Mini Kit (Qiagen; www.qiagen.com) according to the manufacturer’s instructions. Three *BTG*-silent and three *BTG*-active individual plants were used from segregating populations for each mutant. For bisulfite treatment, 200-500 ng of genomic DNA was converted using the MethylEasy Xceed Rapid DNA Bisulphite Modification Kit (Human Genetic Signatures Pty Ltd; North Ryde, Australia; www.geneticsignatures.com) according to the manufacturer’s instructions. The Kismeth plant bisulfite sequencing primer design program (Gruntman *et al.* 2008) (http://katahdin.mssm.edu/kismeth) was used to design degenerate primers that were used to amplify converted DNA from the transgene. Primer locations were selected to selectively amplify the 35SBTG construct promoter and transcriptional enhancer regions. The selected primers designed to amplify portions of the 35S CaMV promoter + *adh1* intron are as follows (R refers to purine; Y refers to pyrimidine):

KM64: 5′-AAAGGAYAGTAGAAAAGGAAGGTGG-3′;
KM69: 5′-CAAACTTTTCRCRCTTRCTAAACAC-3′;
KM73: 5′-CCTCCTTRCAARTTRCAACATTCT-3′;
KM78: 5′-ATYATTGYGATAAAGGAAAGG-3′.

Nested PCR was used to amplify desired products. For the first round of PCR, KM64 and KM69 primers were used. PCR conditions were as follows: 94° for 3 min (1×); 94° for 30 sec, 52° for 1 min, 72° for 1.5 min (30×); 72° for 10 min (1×). 2 µl of first-round PCR product were used as template for the nested PCR reaction using KM73 and KM78 primers. PCR conditions were as follows: 94° for 3 min (1×); 94° for 30 sec, 44° for 1 min, 72° for 1.5 min (30×); 72°C for 10 min (1×). PCR products were gel purified using the Wizard SV Gel and PCR Clean-up System (Promega; www.promega.com), cloned into the pCR4 TOPO TA cloning vector (Invitrogen; www.invitrogen.com) and transformed into TOP10 Chemically competent cells (Invitrogen). Plasmid DNA was isolated using the QIAGEN Plasmid Mini Kit (Qiagen) according to the manufacturers’ instructions. Cloned inserts were verified by PCR using insert specific primers and by restriction digestion using the *Eco*RI enzyme with restriction sites within the pCR4 vector. M13R primers were used for sequencing. The analysis included three individual plants for each of the seven different types of plant (*tgr1 BTG*-s, *tgr1 BTG*-a, *tgr2 BTG*-s, *tgr2 BTG*-sec, *tgr2 BTG*-a, *tgr3 BTG*-s, and *tgr3 BTG*-a). For each individual plant, 10-12 clones were sequenced and subjected to analysis. The Kismeth plant bisulfite sequencing analyzer (Gruntman *et al.* 2008) (http://katahdin.mssm.edu/kismeth) was used for methylation analysis. Clones that appeared to be duplicate representation of the same molecule were identified and removed from analysis (Henderson *et al.* 2010) as these might artificially overrepresent the methylation pattern and bias the data. Because a nonproofreading enzyme was used for PCR amplification, any sequences with more than 0.8% non C/T mismatches in the analyzed region were also removed them analysis. Removing these potentially duplicate or error-containing clones resulted in the evaluation of between 10 and 31 clones for each mutant genotype and BTG expression level that was analyzed. An endogenous unmethylated sequence, Probe A/*Pst*I (Stam *et al.* 2002a), was used as a control to confirm that the retention of cytosines in the sequences did not result from incomplete conversion reactions. The PCR conditions are the same as those described for analysis of methylation in the 35S CaMV promoter of BTG and primers used to amplify this control sequence are:

KM447: 5′-TTGGAAGAGTYAAGAGTGGYAGGTA-3′;
KM 448: 5′-GGATGGATGTAATTAAATAYAGTAG-3′;
KM449: 5′-CATRCATCTCCCTCTCTATCTC-3′;
KM450: 5′-TTCAARCTATACACTRCAACAC-3′.

### Linkage relative to putative maize RdDM components with characterized mutants in maize

The *BTG*-silent lines had been crossed for multiple generations with a stock that is polymorphic relative to the inbred line B73, enabling B73 to be used as the outcross parent for constructing mapping populations. To generate mapping populations, F_1_ seed resulting from crosses of *tgr BTG*-silent plants with B73 were planted, and plants were self-pollinated. Resulting F_2_ seed were planted, and tissue was collected from segregating families. For each mapping population, a minimum of 30 individuals homozygous for the mutant, based on their purple phenotype, were genotyped for a series of SSR markers with known chromosomal locations. SSR markers were selected based on their linkage with genes considered to be potential candidates for the *tgr* mutants (*Mop1*, *Mop2*, *Rmr1*, and *Rmr6*), and that they were polymorphic in the parental lines for the mapping populations. Chromosomal location, names of the linked markers, and primer sequences for each marker are provided in the supporting information (Table S1).

### SSR genotyping

Polymerase chain reaction (PCR) conditions and cycling profile are based on the original protocol established for maize SSR mapping (Sharopova *et al.* 2002). Briefly, PCR reactions for genotyping were as follows: 1× Bioline PCR Buffer (www.bioline.com; as per manufacturer’s instructions); 2.5 mm MgCl_2_; 0.4 mm each dNTP (dATP, dCTP, dGTP, dTTP); 50 ng each forward and reverse SSR primer; 0.3 units Bioline TAQ Polymerase; and 50 ng of genomic DNA template. PCR reactions were brought up to a final volume of 15 µl with sterile H_2_0.

All PCR reactions were performed in a 96-well thin-walled microtiter style plate in a Perkin Elmer thermocycler using the following program: 95° for 1 min, 65° for 1 min, 72° for 1.5 min for one cycle and then decreased 1° per cycle, until the annealing temperature is 55°. The regime is then 95° for 1 min, 55° for 1 min, 72° for 1.5 min, repeated for a total of 30 cycles. After the addition of 5× loading dye (3 µl), 4% Super Fine Resolution agarose (Amresco) gels were loaded with 7.5 µl of each PCR reaction and run with constant voltage of 90-100 V for ∼120 min, which achieved the best resolution. Most maize SSR polymorphism band sizes range between 90 and 150 bp and are well resolved using these conditions that were generally able to detect size differences of 5-10 bp. After running, gels were stained in ethidium bromide and photographed and the resultant pictures evaluated for the F_2_ genotype. All gels contained multiple occurrences of the two parents to the F_1_ as this allowed for greater certainty in scoring the F_2_ progeny.

## Results and Discussion

### The silent *b1* transgene is an effective phenotype for the identification of mutants defective in transgene silencing

The transgene is stably silent in wild-type backgrounds, resulting in plants without observable anthocyanin pigmentation in most tissues (Figure 1B). Based on nuclear run on assays, DNA methylation, and genetic analysis, the transgene is transcriptionally and epigenetically silenced in wild-type plants (McGinnis *et al.* 2006). Although prior work had shown that three genetic factors identified as required for paramutation were also required for maintaining transgene silencing (McGinnis *et al.* 2006), reactivation of *BTG*-silent had not been used for forward genetic screens. As it is likely that transcriptional silencing is dependent upon many proteins, a forward genetic screen for transgene reactivation was conducted to identify additional proteins required for silencing this locus.

For this screen, nontransgenic pollen was mutagenized with EMS and applied to ears of plants that were homozygous or hemizygous for *BTG*-silent. Plants representing the M_1_ and M_2_ generations were visually screened for evidence of loss of transgene silencing associated with dominant or recessive mutations, respectively (Figure 1C). In the M_1_ generation, ∼3000 plants were screened, and all demonstrated a silent transgene phenotype, revealing no dominant nor semidominant mutations.

Within the ∼500 M_2_ families, a total of 10 were observed to segregate plants with dark pigment, indicative of reactivated transgenes, and these were designated *tgr1* through *tgr6* and *tgr8* through *tgr11*. In many M_2_ families, other morphological or chlorophyll pigment phenotypes were observed, consistent with a successful mutagenesis by EMS (Neuffer *et al.* 1997). In most of the putative *tgr* mutants, the plants were very dark, consistent with extensive up-regulation of the transgene (Figure 1C). The *tgr3* mutant was an exception, as it exhibited a unique phenotype consistent with only a modest reactivation of the transgene (Figure 1D).

Chi square (χ^2^) analysis was used to test the fit of the observed values to the expected values for the segregation of recessive mutations. Nine of the ten families demonstrated segregation consistent with the presence of a single, recessive, mutated allele (Table 1). The *tgr9* family segregated significantly fewer individuals than expected for a recessive mutation over multiple generations (Table 2), indicating that this may not be a fully penetrant allele, that there may be reduced transmission of *tgr9*, or reduced viability of *tgr9* homozygotes.

**Table 1:** Segregation of BTG-active plants in M_2_ families

Family	Hypothesis*^a^*	No. of *BTG*-active Plants	No. *BTG*-silent Plants	χ^2^*^b^*
*tgr1*	(1:3)	4	8	0.44
*tgr2*	(1:3)	4	25	1.94
*tgr3*	(1:3)	6*^c^*	18	0.00
*tgr4*	(1:3)	2	8	0.13
*tgr5*	(1:3)	2	8	0.13
*tgr6*	(1:3)	5	16	0.02
*tgr8*	(1:3)	4	7	0.76
*tgr9*	(1:3)	3	28	3.88^*^
*tgr10*	(1:3)	3	7	0.43
*tgr11*	(1:3)	11	19	2.17

^a^ The null hypothesis reflects a ratio consistent with the segregation of a recessive mutation in a population that resulted from self-pollination of a heterozygous individual.

^b^ Chi square tests (χ^2^) were used to estimate the degree of confidence for the hypothesis (*P* = 0.05) for each mutant family.

^c^ In *tgr3* families, *BTG*-active plants exhibit a subtle pigmentation phenotype (Figure 1D).

^*^ Significant difference.

**Table 2:** Segregation of transgene activity in the M_3_ generation

Mutation	Hypothesis*^a^*	No. of *BTG*-active Plants	No. of *BTG*-silent Plants	χ^2^*^b^*
*tgr1*	(1:3)	8	21	0.12
*tgr2*	(1:3)	35*^c^*	22	40.9^*^
	(3:1)	35*^c^*	22	5.60^*^
	(1:2:1)	20; 15*^d^*	22	12.9^*^
	(7:9)	35	22	7.125^*^
*tgr3*	(1:3)	35*^e^*	24	40.76^*^
	(3:1)	35*^e^*	24	7.70^*^
	(7:9)	35*^e^*	24	5.57^*^
*tgr6*	(1:3)	2	9	0.27
*tgr9*	(1:3)	6	42	4.00^*^
*tgr10*	(1:3)	1	7	0.667
*tgr11*	(1:3)	10	30	0.00

^a^ The null hypothesis reflects a ratio consistent with a recessive mutation (1:3), dominant mutation (3:1), or semidominant mutation (1:2:1) in a population that resulted from self-pollination of a heterozygous individual or a ratio consistent with two recessive mutations (7:9) segregating in a population that resulted from the self-pollination of an individual heterozygous for both mutations.

^b^ Chi square tests (χ^2^) were used to estimate the degree of confidence for each hypothesis (*P* = 0.05).

^c^ For this value, dark (n = 20) and sectored (n = 15) individuals are combined and considered as one phenotypic category.

^d^ For these values, dark and sectored individuals were grouped into different categories, and the sectored phenotype exhibited by 15 individuals was assumed to be associated with heterozygous mutations.

^e^ In *tgr3* families, *BTG*-active plants exhibit a subtle pigmentation phenotype (Figure 1c).

^*^ Significant difference.

Previous work demonstrated that mutations in two genes that encode maize orthologs of the Arabidopsis RdDM pathway resulted in reactivation of *BTG*-silent (McGinnis *et al.* 2006); *mop1* that encodes an RNA-dependent RNA polymerase (Alleman *et al.* 2006), and *rmr1* that encodes a protein related to chromatin remodeling proteins (Hale *et al.* 2007). Maize mutants for the orthologs of every known component of this pathway in Arabidopsis have not yet been identified, and more than one silencing pathway could function at a given locus. Thus, the putative mutants from this screen might represent mutations in uncharacterized genes, new mutations in the maize RNA-directed transcriptional gene silencing pathway, or mutations in genes encoding proteins previously implicated in other gene silencing pathways.

### Two mutants exhibit unique phenotypes and segregation patterns in the M_3_ generation

Segregating M_2_ families included *BTG*-silent and *BTG*-active plants, identified as green and purple plants, respectively (Figure 1). The *BTG*-silent individuals were potentially heterozygous for the mutation or homozygous for the wild-type allele, while the BTG-active individuals were potentially homozygous for a recessive allele. To test these hypotheses and to analyze the genetic behavior of the newly discovered putative mutants in further generations, *BTG*-silent, M_2_ individuals were self–pollinated, and the activity of the transgene was observed in the M_3_ progeny. These tests were completed for seven of the mutants because *tgr4*, *tgr5*, and *tgr8* proved difficult to propagate due to reduced seed set and low germination frequencies. With two exceptions discussed below, the segregation ratios observed in the M_3_ generation were similar to those observed in the M_2_ generation (Table 2).

**Figure 2:**
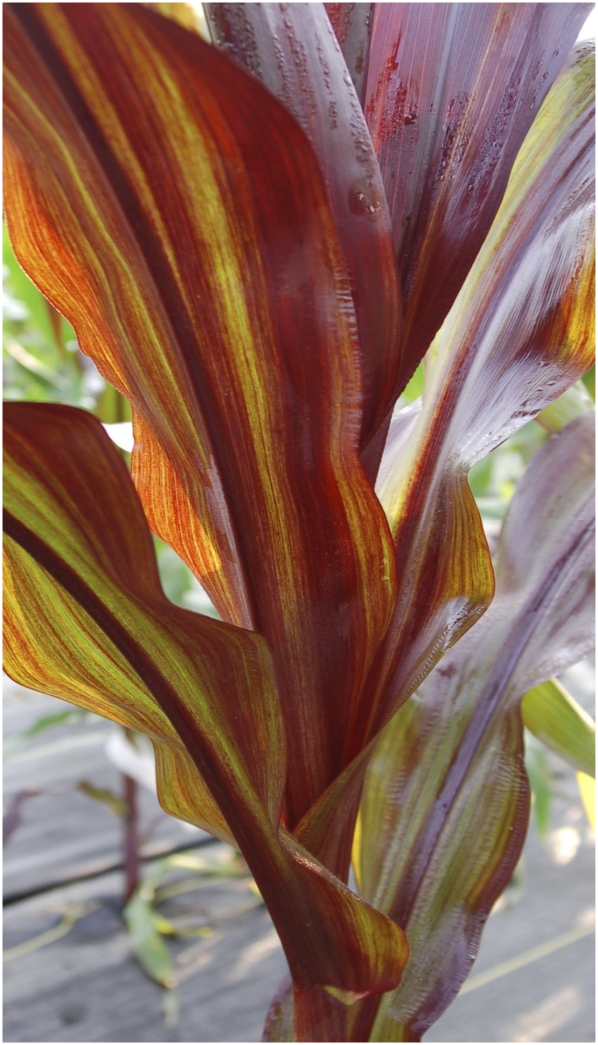
Sectored phenotype observed in *tgr2* families. In addition to a phenotype consistent with a high level of transcriptional activity, some plants in *tgr2*-derived lines exhibit a sectored phenotype consistent with transgene silencing in some sectors of affected individuals. This was an exceptional phenotype first observed in segregating M_3_ families generated by self-pollinating a heterozygous individual in the M1 generation (Table 2).

For *tgr2*, a reduced number of green plants and the appearance of a new phenotypic class with sectors (Figure 2) did not fit the simple hypothesis for segregation of a single recessive mutation (Table 2). This sectored phenotype (*BTG*-sec) is consistent with the transgene only being reactivated in some cell lineages, or with the transgene becoming resilenced in a subset of cells in the presence of an activating mutation. While the presence of the third phenotypic class might suggest *tgr2* is semidominant, with heterozygous individuals exhibiting the sectored phenotype and homozygous mutant individuals exhibiting the confluently, darkly pigmented phenotype, the segregation ratio did not match that expectation based on χ^2^ analysis, nor the hypothesis that *tgr2* is a dominant mutation. Furthermore, the M_1_ generation that produced *tgr2* did not exhibit a *BTG*-active phenotype, which is inconsistent with the hypothesis that *tgr2* is a dominant or semidominant allele. The *tgr2* data also did not match the expectation for two independent, recessive mutations (Table 2). This suggests that the *tgr2* mutation has unexplained but complex genetic characteristics. Simple genetic analysis of these traits is further complicated by the persistence of the *BTG*-active phenotype after the inducing mutation has been segregated away. This characteristic of *tgr2* is described in more detail below.

In *tgr3* families, fewer green plants than expected for a recessive mutation are observed. Similar to *tgr2*, in *tgr3* families, the segregation ratios in the M_3_ generation do not match a 1:3, 3:1, or 7:9 ratio (Table 2), which would be consistent with recessive, dominant, or two independent mutations, respectively. As discussed for *tgr2*, this result may be indicative of complex genetic behavior of the *tgr3-1* allele.

Based upon their diverse genetic behaviors and ease of propagation, *tgr1*, *tgr2*, and *tgr3* were selected for further investigation and subjected to more extensive molecular and genetic analysis.

### Transgene reactivation is heritable in *tgr2* lines

**Figure 3:**
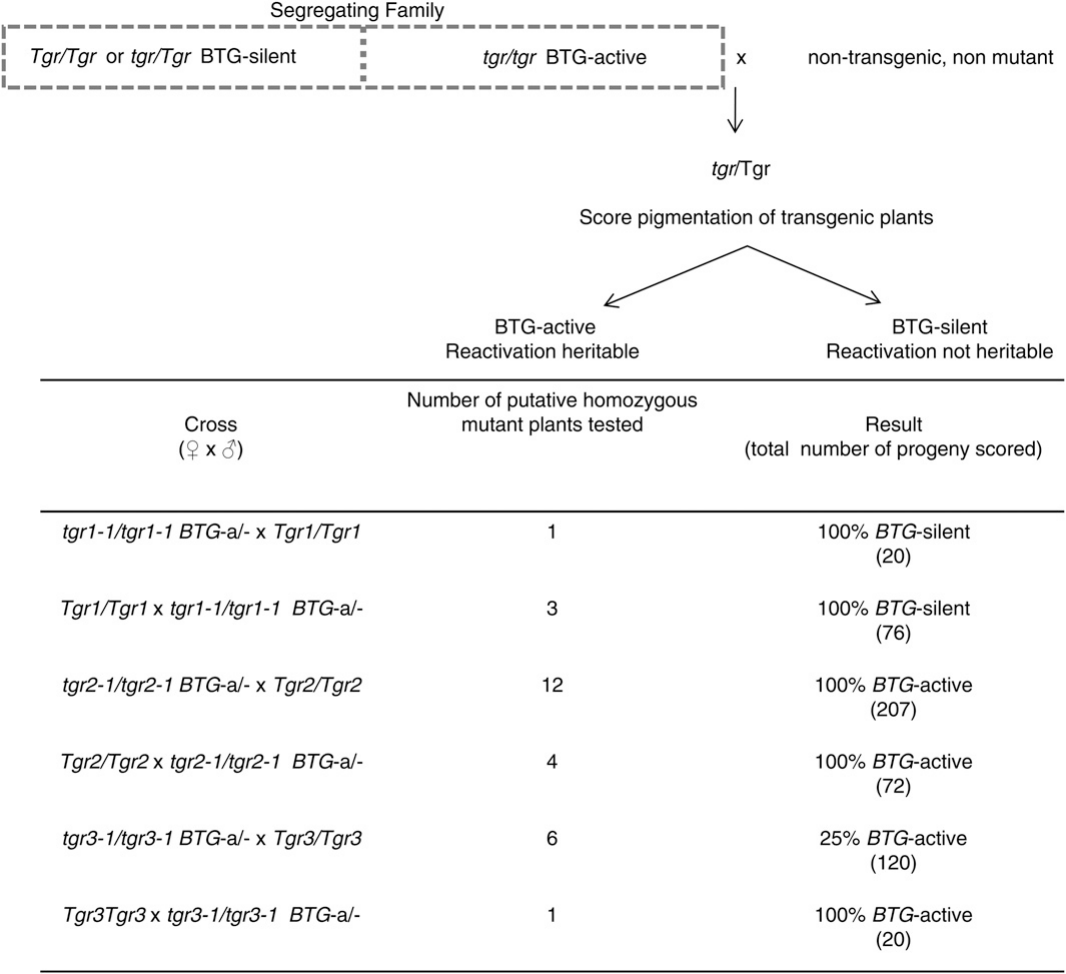
Crossing strategy used for testing the heritability of transgene expression in *tgr1*, *tgr2*, and *tgr3*. Plants with active transgenes were outcrossed for one generation with nontransgenic, nonmutant plants to observe if the transcriptional activity would persist through meiosis and the reintroduction of wild-type proteins in the next generation. In the crosses the female parent is listed first and the male parent listed second. Reciprocal crosses varying whether the transgene was transmitted through male or female were done to compare heritability through both parents.

It was previously demonstrated that transgene activity persisted after segregating away the *mop1* and *rmr2* mutants (McGinnis *et al.* 2006). This was referred to as heritability of activation, and this activation became increasingly stable after subsequent generations of being maintained in wild-type backgrounds. Heritability of activation was tested in *tgr1*-, *tgr2*-, and *tgr3*- derived lines (Figure 3). For this assay, *BTG*-active plants in segregating families were crossed with nontransgenic, nonmutant genetic stocks, and transgenic plants were observed for pigmentation. Transgenic progeny from *tgr2* parents were all darkly pigmented, indicating that the transcriptionally active state persisted through meiosis and was heritable, and that the transgene was not resilenced in the presence of the wild-type *Tgr2* allele. This is the same phenotype previously observed for the *mop1* and *rmr2* mutants.

In *tgr1*-derived lines, the progeny of outcrossed plants were all green, indicating that silencing was efficiently restored upon introduction of the wild-type allele. The *tgr1* phenotype is similar to that reported for a *rmr1* mutant in previous studies (McGinnis *et al.* 2006). For lines derived by outcrossing *tgr3* individuals with active transgenes, heritability was observed at a relatively low level compared with *tgr2*-derived lines. Thus, the *tgr* mutants collectively present a full spectrum of epigenetically heritable and nonheritable phenotypes for in depth studies on transmission of epigenetic information from one generation to the next.

### DNA methylation in *tgr1* and *tgr2* mutants correlates with transgene reactivation

Consistent with their involvement in the RdDM pathway, loss of Mop1, Rmr1, and Rmr2 resulted in reactivation of the *BTG*-silent correlated with a reduction in cytosine methylation of the 35S CaMV promoter within the transgene (McGinnis *et al.* 2006). To determine whether transgene reactivation in the newly discovered mutants correlated with changes in DNA methylation, cytosine methylation of the 35S CaMV promoter in the transgene was determined using sequence analysis of PCR products from bisulfite converted genomic DNA.

The *adh1* intron was used to specifically amplify the 35S CaMV promoter directly driving *b1* expression (Figure 1A) and not the 35S promoter associated with the *bar* transgene that was used for selection when it was cotransformed (McGinnis *et al.* 2006). In nonmutant *BTG*-silent plants (six clones), 76.39% (CG, 88.33%; CHG, 80.55%; CHH, 73.10%) of the cytosines were methylated within the analyzed portion of the 35S CaMV promoter, which included 220 base pairs and a total of 60 cytosine residues. Approximately 410 base pairs of the immediately adjacent *adh1* intron of the transgene was also analyzed; this region was consistently unmethylated in converted DNA extracted from *BTG*-active and *BTG*-silent plants (data not shown).

Methylation within the 35S CaMV promoter region in *BTG*-active plants from segregating *tgr1*, *tgr2*, *and tgr3* families were compared with *BTG*-silent plants within the same families. In combination, these three mutants allowed for analysis of plants demonstrating a range of pigmentation, phenotypic heritability, and inheritance patterns.

**Figure 4:**
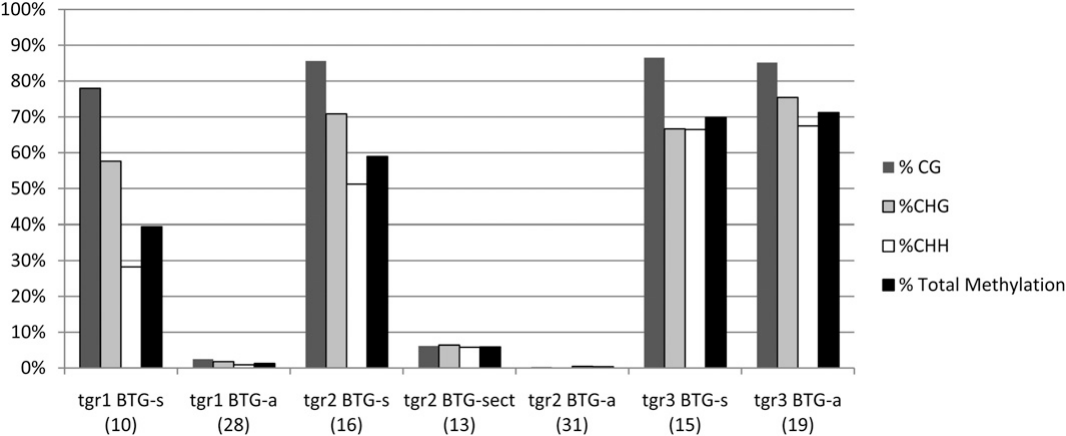
Methylation of the 35S CaMV promoter in three *tgr* mutants. Bisulfite sequencing was used to determine whether there were differences in CG, CHG, and CHH methylation of the 35S promoter in *tgr BTG*-active (*tgr BTG*-a) plants for *tgr1*, *tgr2*, and *tgr3* relative to control siblings carrying *BTG*-silent (*BTG*-s). The *tgr2* mutation includes an additional sectored phenotype (Figure 2) denoted as *tgr2 BTG*-sec. The level of methylation is reported as the percentage of total cytosines in the 35S CaMV promoter exhibiting methylation. The number of clones for each genotype is indicated in parentheses, the analyzed region represents a total of 60 cytosines.

In *tgr1* and *tgr2* mutants, the *BTG*-active plants were hypomethylated when compared with their *BTG*-silent siblings in all methylation contexts (Figure 4), which suggests that Tgr1 and Tgr2 may function in a gene silencing pathway that is associated with cytosine methylation in the promoters of regulated genes. This is consistent with observations of *mop1*, *rmr1*, and *rmr2* mutants (McGinnis *et al.* 2006). Extensive hypomethylation was also observed in *tgr2 BTG*-sec individuals, although some methylation (<10%) is apparent in these plants. In contrast, promoter methylation levels in *BTG*-silent and *BTG*-active appear to be similar in *tgr3* families. In *tgr3* mutants, the low level of BTG activation could mean that there is a slight up-regulation of transcriptional activity in the presence of cytosine methylation, but it is also possible there is a very modest change in methylation that is below the sensitivity level for the detection technique. Further investigation into this relationship may yield additional insight into the correlation between DNA methylation and transcriptional activity.

### *tgr1* is linked to *rmr6*

The loss of epigenetic gene silencing characteristic of the mutants identified in this screen is similar to the phenotypes used to identify *mop1*, *mop2*, rmr1, and *rmr6* mutants (reviewed by Arteaga-Vazquez and Chandler 2010). This phenotypic similarity may be an indication that the *tgr* mutants represent alleles of these cloned genes. Similar to the phenotypes previously reported for *rmr1* mutants (McGinnis *et al.* 2006), *tgr1* exhibited DNA hypomethylation, but a lack of meiotic heritability of transcriptional reactivation of BTG. The persistence of transgene activity into the next generation after outcrossing as demonstrated by *tgr2-1*, *rmr2-1*, and *mop1-1* confounds allelism analysis by complementation analysis for some mutants, so a molecular approach was used to examine linkage of the *tgr* mutants with genes with previously sequenced mutations, including *Mop1*, *Mop2*, *Rmr1*, and *Rmr6*. Cloning and sequencing of the *rmr2-1* mutation has not been published.

To test for linkage of *tgr1* with sequenced mutations, a total of 36 *tgr1 BTG*-active individuals were analyzed with SSR markers tightly linked to cloned genes associated with epigenetic gene regulation in maize, *mop1*, *mop2*, *rmr1*, and *rmr6*. For three genes no linkage was identified (Table 3). For an *Rmr6*-linked marker, the *tgr1* parental allele was overrepresented, suggesting that the gene bearing the *tgr1* mutation lies on chromosome 1, and is linked to *rmr6*. The presence of heterozygous individuals in this small population suggests that *tgr1* is not an allele of *rmr6*. Several developmental abnormalities have been described for *rmr6-1* and *rmr6-2* alleles, including abnormal leaf polarity and male inflorescence development (Parkinson *et al.* 2007), none of which have been observed in segregating *tgr1* families. Further mapping and complementation tests will be required to determine the molecular identify of *tgr1*.

**Table 3:** *Tgr1* is linked to *Rmr6*

Linked Gene	SSR Locus Name	Number of Individuals Homozygous for Tgr Parental Allele	Number of Heterozygous Individuals	Number of Individuals Homozygous for B73 Parental Allele
*Mop1* (chromosome 2)	UMC1465	4	10	20
*Mop2* (chromosome 2)	UMC2403	6	19	8
*Rmr1* (chromosome 6)	UMC2320	7	18	8
*Rmr6 (chromosome 1*)	BNLG1025	26	12	0

There is also some evidence of an overrepresentation of the B73 allele of a chromosome 2 localized marker in this population. Segregation distortion has been reported in other maize mapping populations, and the effected loci seem to vary in a population-dependent manner (Sharopova *et al.* 2002). The presence of a higher number of individuals with the B73 allele than the *tgr* parent allele for this marker may either be caused by observation of segregation in a relatively small population or reflect an example of segregation distortion in the population.

### *tgr2* is not linked to cloned mutations with similar phenotypes

In addition to the characteristic loss of epigenetic silencing phenotype, *tgr2* exhibited DNA hypomethylation and heritability of transgene reactivation, which are phenotypes that have been previously reported for *mop1* and *rmr2* mutants (McGinnis *et al.* 2006). *Tgr2* linkage was analyzed in a similar manner to that described for *tgr1* to determine if these common phenotypes were an indication of allelism between *tgr2* and previously sequenced mutations.

To test linkage of the *tgr2 BTG*-active phenotype with the candidate genes, 38 individuals were analyzed. For each analyzed gene, populations of purple plants exhibiting the *tgr2*-related *BTG*-active phenotype were not genotypically biased toward the *tgr2* parent allele for that locus (Table 4), suggesting a lack of linkage between the phenotype and the genetic locus being tested. This suggests that although *tgr2* shares many phenotypes with previously characterized mutations in these genes, it is unlikely to represent an allele of one of these genes. For some markers, the B73 allele was detected in more individuals than the *tgr2* parental allele, indicating an unusual segregation pattern and potential segregation distortion.

**Table 4:** *Tgr2* is not linked to previously cloned components of the maize RdDM pathway

Linked Gene	SSR Locus Name	Number of Individuals Homozygous for Tgr Parental Allele	Number of Heterozygous Individuals	Number of Individuals Homozygous for B73 Parental Allele
*Mop1* (chromosome 2)	UMC1465	6	13	18
*Mop2* (chromosome 2)	UMC2403	6	13	20
*Rmr1* (chromosome 6)	UMC2320	9	27	4
*Rmr6* (chromosome 1)	UMC1035	7	17	15

### *tgr3* is not linked to *mop1*, *mop2*, *rmr1*, or *rmr6*

While more subtle than that observed for other mutants (Figure 1D), the *tgr3* phenotype is indicative of a reduction in the epigenetic silencing at BTG. While the BTG-a phenotype is notably distinct from that observed for other mutants, loss of silencing is consistent with the phenotype of *mop1*, *rmr1*, and *rmr2* mutants. Thus, linkage was tested for *tgr3* as described for *tgr1* and *tgr2* (Table 5). In *tgr3 BTG*-active plants, the parental mutant alleles were not overrepresented and the B73 parental alleles were not underrepresented, demonstrating that *tgr3* is not an allele of *mop1*, *mop2*, *rmr1*, or *rmr6*. The B73 allele appeared to be overrepresented for a marker on chromosome 1; segregation distortion toward the B73 allele was reported for several markers in this chromosomal region in an intermated B73 / Mo17 population (Sharopova *et al.* 2002).

**Table 5:** *Tgr3* is not linked to previously cloned components of the maize RdDM pathway

Linked Gene	SSR Locus Name	Number of Individuals Homozygous for Tgr Parental Allele	Number of Heterozygous Individuals	Number of Individuals Homozygous for B73 Parental Allele
*Mop1* (chromosome 2)	UMC1465	7	14	18
*Mop2* (chromosome 2)	UMC1823	6	21	12
*Rmr1* (chromosome 6)	UMC2320	10	14	8
*Rmr6 (chromosome 1*)	UMC2560	3	13	20

## Conclusions

These results demonstrate that reactivation of the silent *35SBTG* transgene in maize is an effective epigenetic phenotype for use in a forward genetic screen. The application of this screen led to the identification of multiple mutants, which exhibit some distinct phenotypes relative to one another and to previously identified maize mutants that can reactivate the silent transgene. Mapping studies suggest that *tgr2* and *tgr3* do not represent alleles of previously cloned genes with similar phenotypes in maize, while *tgr1* resides on chromosome 1 and is linked to *rmr6*. Further, *tgr2*- and *tgr3*-derived lines exhibited persistent transgene reactivation after the reactivating mutation had been segregated away, meaning that *BTG*-silent provides a useful platform for studying heritable changes in gene expression in plants. Additional study of these mutants should yield further insight into epigenetic gene regulation.

## Supplementary Material

Supporting Information
