# Supplementary material for: Identification of Epigenetic Regulators of a Transcriptionally Silenced Transgene in Maize
Source: G3 (Bethesda). 2011 Jun 1;1(1):75–83. doi: 10.1534/g3.111.000232 (PMC3276119; doi:10.1534/g3.111.000232)
Supplement: Supporting Information [file supp_1_1_75__index.html]

Supporting Information 

# Identification of Epigenetic Regulators of a Transcriptionally Silenced Transgene in Maize

## Supporting Information for Madzima *et al.*, 2011

**Files in this Data Supplement:**

- Table S1 - SSR markers used for linkage analysis (PDF, 44 KB)
