# Supplementary material for: Identification of Epigenetic Regulators of a Transcriptionally Silenced Transgene in Maize
Source: G3 (Bethesda). 2011 Jun 1;1(1):75–83. doi: 10.1534/g3.111.000232 (PMC3276119; doi:10.1534/g3.111.000232)
Supplement: Supporting Information [file supp_1.1.75_TableS1.pdf]

Table S1   SSR markers used for linkage analysis

Gene	Location[a]	Marker[b]	Forward primer[c]	Reverse primer[c]
MOP1	2.04	UMC1579	AAGATCAGCTAGCGAGAGAAGCAA	AGGAGGTCAGTGCTGCAGGT
		UMC2032	TCTATCATTCGAGTCAAGAAGCCA	AAAAGAAGACGGATTTCTTCGGAC
		UMC1541	TGCAATCATTCTTCCATGTCTTGT	GCACAATCTGCTGGTACTTCTTGA
		UMC1465	GTACTTCAAAGCACATGAATGCGA	AGTCTCGACTGCCAATTTACCAAA
MOP2	2.02	UMC1265	GCCTAGTCGCCTACCCTACCAAT	TGTGTTCTTGATTGGGTGAGACAT
		UMC2403	TTCGTATCTTAGCTGGTCTTTGGC	AGTCAGTCGATCGTACACAGTCCA
		UMC1961	GGATGAGAATTGAGAAAGAGCCAA	CATGTCCATACACAGTCATACACTGC
		UMC1823	AAAGCCTTACTGTTATTAGGCTAGGCA	AGAAAACCAGCCCCAGATGTTC
RMR1	6.05	UMC2321	GACTGTAGAGTCGCGGGTGTATTT	TCCCATCTCATGACCAGTACAAAA
		UMC2320	TAACCTCTAGCAGCACATGCACAC	GAGAGTTTTATCAGCAGCAAAGCC
		UMC2065	CAAGGTTCGGTCCTTCTTCTCC	GACACCTCGTCGTCGGTCAC
		UMC2040	GAGACACAGGAACAGAACCCTCTC	GAGACATCTCGACACCTCTTGTGA
RMR6	1.06	UMC1035	CTGGCATGATCACGCTATGTATG	TAACATCAGCAGGTTTGCTCATTC
		UMC2568	GACGAGTTGTCGTCGCATCTT	GTCTCTTCTTCGACATGGCCTTC
		UMC2560	TACAACAGTTCTACACCCCGTCCT	TAATGCATGGGACGAGTAGGTTTT
		UMC1709	ATACGTAGCACCTCCAGGTAGCAG	CAGGTACAACGGATGAGGCAG
		UMC1849	TCCTTGTTGAAGATTTTATTTCTGCT	GGCTTTAAGTGATGCTCAAACGTA
		UMC2217	ATGTCCCATTTACCAAGGTCTGTG	AAGACCTGTTCGTCAACCATGTTC
		UMC2390	GAAATGGCAGGGAAACTTGTTTAT	AAGAGGCAAGCAAGTGTACAGTGA
		UMC1144	ATGGCCCACTCATCATATCTCTGT	TGTGTTGATTAGCAGCGGATAAAA

[a]Chromosomal locations of candidate genes indicated by chromosome and bin (chromosome number. bin number).

[b] Marker names as described by Sharapova et al. (2002), additional information on these markers available at www.maizegdb.org.

[c] Primers sequences in the 5’ to 3’ orientation.
